# Evaluating and implementing temporal, spatial, and spatio-temporal methods for outbreak detection in a local syndromic surveillance system

**DOI:** 10.1371/journal.pone.0184419

**Published:** 2017-09-08

**Authors:** Robert W. Mathes, Ramona Lall, Alison Levin-Rector, Jessica Sell, Marc Paladini, Kevin J. Konty, Don Olson, Don Weiss

**Affiliations:** New York City Department of Health and Mental Hygiene, Queens, New York, United States of America; Fundacao Oswaldo Cruz, BRAZIL

## Abstract

The New York City Department of Health and Mental Hygiene has operated an emergency department syndromic surveillance system since 2001, using temporal and spatial scan statistics run on a daily basis for cluster detection. Since the system was originally implemented, a number of new methods have been proposed for use in cluster detection. We evaluated six temporal and four spatial/spatio-temporal detection methods using syndromic surveillance data spiked with simulated injections. The algorithms were compared on several metrics, including sensitivity, specificity, positive predictive value, coherence, and timeliness. We also evaluated each method’s implementation, programming time, run time, and the ease of use. Among the temporal methods, at a set specificity of 95%, a Holt-Winters exponential smoother performed the best, detecting 19% of the simulated injects across all shapes and sizes, followed by an autoregressive moving average model (16%), a generalized linear model (15%), a modified version of the Early Aberration Reporting System’s C2 algorithm (13%), a temporal scan statistic (11%), and a cumulative sum control chart (<2%). Of the spatial/spatio-temporal methods we tested, a spatial scan statistic detected 3% of all injects, a Bayes regression found 2%, and a generalized linear mixed model and a space-time permutation scan statistic detected none at a specificity of 95%. Positive predictive value was low (<7%) for all methods. Overall, the detection methods we tested did not perform well in identifying the temporal and spatial clusters of cases in the inject dataset. The spatial scan statistic, our current method for spatial cluster detection, performed slightly better than the other tested methods across different inject magnitudes and types. Furthermore, we found the scan statistics, as applied in the SaTScan software package, to be the easiest to program and implement for daily data analysis.

## Introduction

In the autumn of 2001 the New York City (NYC) Department of Health and Mental Hygiene (DOHMH) established an emergency department (ED) syndromic surveillance system to detect and track disease outbreaks and illness in the city. We described this system in 2004 [1], along with an initial evaluation of the spatial scan statistic, the aberration detection method used to detect spatial clusters of disease [2]. In the years since implementing these early systems, there has been a broadening of the practice of syndromic surveillance to include uses as diverse as the monitoring of animal and human related injuries [3,4], tattoo related infections [5], seasonal and pandemic influenza surveillance [6–8], and monitoring of the health effects of pollen and particulate matter air quality [9]. There have also been significant developments in the field of syndromic surveillance, related to the adoption of standardized data technology, i.e. Health Level 7 (HL7) messaging [10], and modifications to existing statistical methods for disease cluster detection [11–13].

A number of studies have investigated temporal [12,14–19] and spatial/spatio-temporal [11,20–22] method performance, with a focus on detecting large simulated outbreaks within simulated and actual syndromic data. While the majority of these studies have found good sensitivity with detecting large, rapid increases in cases, most have been less successful with finding smaller outbreaks, which are more likely to occur in practice. Furthermore, these studies show that using as much historical data that is available can be beneficial [20], as well as testing methods on multiple outbreak types and data streams of differing baseline counts [15,20], including varying day of week and seasonal patterns [21]. Finally, adjusting for these temporal patterns can significantly improve performance [14,20,23]. However, few of these studies compared performance between multiple spatial/spatio-temporal methods.

Our goal in this study was to evaluate alternative statistical methods for use in ED syndromic surveillance and compare their performance to our current temporal and spatial methods. Thus, methods were evaluated based on their ability to detect simulated injects in actual syndromic data, as well as their ease of development and implementation into a daily surveillance system in a state or local health department.

## Methods

### Ethics statement

We did not submit this project to the NYC DOHMH Institutional Review Board, as the existing data are collected as part of routine public health surveillance, could not be linked to individuals, and were analyzed anonymously.

### Data collection

At the time of this study, data from 51 hospital EDs in NYC, comprising approximately 98% of all annual ED visits, are transmitted electronically to DOHMH daily in either flat files via file transfer protocol or as HL7 messages. Variables transmitted include date and time of patient visit, medical record number, patient demographics including age, sex, and residential ZIP code, the reason for visit or chief complaint, how the patient arrived at the ED, and discharge diagnosis (International Classification of Diseases Code version 9 and 10). For our syndrome classifier, a text processing algorithm scans the free-text chief complaint field to identify keywords indicative of a syndrome, such as “cough”, “sore throat”, and “fever” for influenza-like illness, while accounting for misspellings, abbreviations, and negations. Each visit is then categorized into one of several syndromes, including but not limited to, asthma, respiratory, diarrhea, vomit, fever/influenza, and influenza-like illness, as previously described [2,7]. A more complete description of our keywords and syndromes is found in Heffernan, et al. [2]. All text processing and data management is done using SAS (version 9.4, SAS Institute Inc, Cary, NC).

### Training and spiked datasets

**Fig 1:**
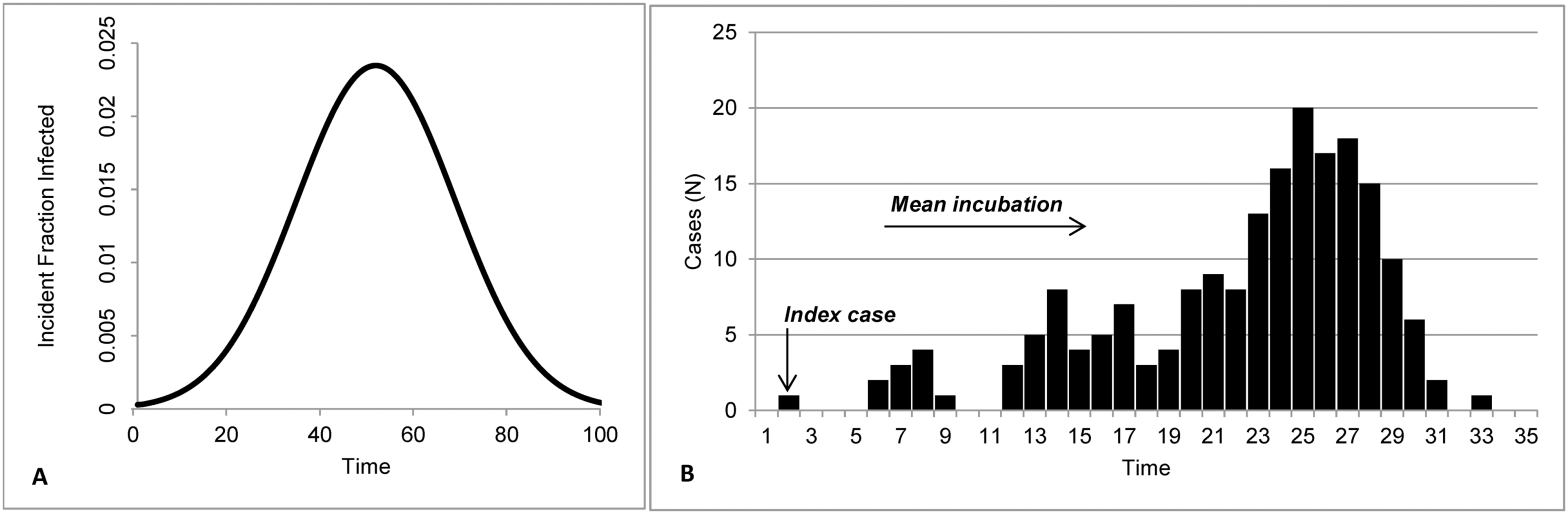
Epidemic curves for (A) point-source exposure and (B) propagated transmission.

We used daily NYC syndromic data from 2007–2009 as a training dataset to fine tune the models. Each model was run on these data and final parameters were chosen. Then, to test the outbreak detection capabilities of the methods, we created datasets containing a baseline of real syndromic data spiked with one simulated inject. Each baseline contained 730 days, starting January 1, 2010 and ending December 31, 2011. Simulated injects were created based on four characteristics: magnitude, epidemic curve shape, duration, and number of ZIP codes affected. Inject magnitude *M* was based on Fricker, et al. [14] and defined for each season (winter, spring, summer, and autumn) as small (*M* = [*μ* + 3*σ*]/4), medium (*M* = [*μ* + 3σ]/2), and large (*M* = [*μ* + 3σ]) where *μ* and *σ* are the mean and standard deviation, respectively, of the syndrome during the season-specific study period. The total number of injected cases was then distributed over time according to three shapes of epidemic curve: single-day spike, point source exposure, and propagated transmission. A single-day spike is composed of a single day in which the count of a particular syndrome exceeds its expected value. The inclusion of a one-day outbreak in our analysis reflects knowledge of similar prior disease outbreaks in NYC and elsewhere. A point source exposure is based on a single time-limited exposure, with cases distributed according to a simple bell-shaped epidemic curve and following the analytic expression to calculate the cumulative fraction of infected cases: F(t) = 1/(1+exp(-0.1×t)×((1–0.01)/0.01)) (Fig 1A) [24]. Three durations were considered: 3 days, 5 days, and 15 days. A similar approach was applied to the empirical frequency distribution used for the propagated transmission. The propagated transmission reflects person-to-person infection, where the syndrome count above expected is based on communicability and incubation period (Fig 1B) [24]. The cumulative fraction of infected cases for each day *d* of an outbreak of duration *D*, as represented by: *t* = *d*×32/*D*. The duration of a propagated transmission was randomly selected as a number of days between 15 and 32 days; we limited duration to approximately one month mainly for practical purposes. The number of cases for each day of the inject period was computed using Monte Carlo simulation and the three cumulative epidemic curve types [15]. For an inject of magnitude *M*, a series of *M* random numbers p^l^ uniformly distributed between 0 and 1 were generated and each of the *M* inject cases was assigned to the day *d* such that F(*d*-1) < p^l^ ≤ F(*d*). The result is a specified number of cases for each day of the outbreak {*M(d)*, *d = 1*,…,*D*}, where *D* is the total number of days in the outbreak, such that:

$$M=\overset{D}{\underset{d=1}{\sum }}M{\left(d\right)}_{\;}$$

Once the *M* cases were temporally distributed among the *D* days of the inject period, they were then spatially distributed among ZIP codes using Monte Carlo simulation. The allocation of cases to ZIP codes was straightforward if the inject concerned a single ZIP code. For clusters of *S* ZIP codes (2 ≤ *S* ≤ 10; given the kernel ZIP code and cluster size S, other members of the cluster were selected as the (S-1) ZIP codes that are the closest to the kernel ZIP code based on Euclidean distances between their geographical centroids) and citywide outbreaks (all ZIP codes in NYC), the proportion of the total number of visits recorded on each day *d* was computed for each ZIP code *z* which is a member of that cluster. We limited the maximum number of ZIP codes in a cluster to 10. The *S* NYC ZIP codes were then ranked according to their share of the total number of visits and a cumulative frequency distribution F_d_(*z*) was built. To allocate the *M(d)* cases simulated on the *d*-th day, a series of *M(d)* random numbers p^l^ uniformly distributed between 0 and 1 were generated and each of the *M(d)* cases was assigned to the ZIP code *z* such that F_d_(*z*-1) < p^l^ ≤ F_d_(*z*). The result is a specific number of injected cases for each day of the outbreak and each ZIP code {*M(d;z)*, *d = 1*,…,*D; z = 1*,…,*S*} such that:

$$M\left(d\right)=\overset{S}{\underset{z=1}{\sum }}M{\left(d;z\right)}_{\;}$$

These injects were randomly inserted within the two year baseline while ensuring each season had an equal number of injects to account for seasonality. Characteristics of the average daily counts of the different syndromic baselines, before the simulated injections were inserted, are described in Table 1.

**Table 1:** Daily count distributions of tested syndrome baselines.

Syndrome	Minimum	Maximum	Mean	Standard deviation
**Temporal**				
Diarrhea	65	309	144	42
Fever/Influenza	306	1093	563	113
Influenza-like illness	51	555	187	87
Respiratory	349	1528	793	218
Vomit	147	525	287	66
**Spatial (ZIP Code)**				
Respiratory	0	48	4	5

In summary, a total of 300 citywide datasets, ranging in magnitude of 1 to 343 excess daily cases, and 120 spatial datasets, ranging in magnitude of 1 to 26 daily excess cases, were created. The characteristics of the simulated injections for the temporal analysis is given in Table 2.

**Table 2:** Characteristics of simulated temporal injects, daily counts.

Syndrome	Min	Max	Mean	Number of injects
Diarrhea	1	86	6	60
Fever/Influenza	1	315	17	60
Influenza-like illness	1	205	10	60
Respiratory	1	343	20	60
Vomit	1	110	7	60

Because computational intensity was significantly higher for the spatial/spatio-temporal methods, we inserted injects into only one syndrome in order to reduce overall processing time (Table 3).

**Table 3:** Characteristics of simulated spatial injects, daily counts.

Respiratory	Mean number of cases per ZIP (range)	Mean duration of outbreak (in days)	Mean number of ZIPs	Number of injects
Overall	2 (1–14)	9.3	2.9	120
Single day	3 (1–14)	1	2.7	24
Point source	2 (1–11)	7.6	2.7	72
Propagated	2 (1–13)	22.9	3.7	24

### Method selection

The DOHMH ED syndromic surveillance system at study onset used the SaTScan temporal and spatial scan statistics [25] to detect daily citywide increases and spatial clusters, by hospital and residential ZIP code, for the aforementioned syndromes.

A search was conducted in PubMed, Google Scholar, and ISI Web of Science to identify alternative methods, using keywords (e.g., syndromic surveillance, statistical method, aberration detection, etc.), and resulting manuscripts were categorized into general methods (e.g., Control Chart, Bayesian, Time Series, etc.). Analysts in the Syndromic Surveillance Unit read each manuscript and completed a survey, scoring each method by the following criteria: published in peer-review literature, no license required, able to adapt to systematic variations typically found in syndromic data (e.g., day of week patterns), ability to adjust baselines, ability to handle count/ratio data, and feasibility to be coded in available, commonly used software, such as SAS or R. Methods that met all criteria were then selected for final review and presented to the project’s voluntary Advisory Board (a group of experts in Public Health, Epidemiology, and Statistics, all of whom have experience in syndromic surveillance) for additional consideration. The complete list of methods evaluated, including those we use in our current system, are shown in Table 4.

**Table 4:** Candidate methods for evaluation.

Temporal	Spatio-temporal
Autoregressive integrated moving average (ARIMA)	Bayesian space-time regression
Cumulative sum control chart (CUSUM)	Generalized linear mixed model (GLMM)
Generalized Linear Model (GLM)	Space-time permutation scan statistic
Holt-Winters exponential smoother	Spatial scan statistic
Modified EARS C2	
Temporal scan statistic	

### Method implementation

We fit six temporal and four spatial and spatio-temporal methods to a training dataset containing real NYC syndromic data from 2007–2009. Each model was implemented as it would be for daily syndromic surveillance use and the decisions necessary for implementation reflected this goal. In the following section, we briefly describe each of the models.

### Temporal methods

#### Modified EARS C2

We first evaluated a modified version of the C2 algorithm. The standard C2 method [26] uses seven days of daily syndrome counts to estimate a baseline mean and standard deviation. To determine if the previous day’s counts are unusual, a test statistic is calculated by subtracting the baseline mean from the previous day’s counts and dividing by the baseline standard deviation. An alert is generated when the test statistic is ≥3 standard deviations. Four modifications as suggested by Tokars, et al [12] were incorporated: (1) stratification of baseline days by weekdays versus weekends; (2) lengthen the normal 7-day baseline to 14 and 28 days; (3) adjust for total visits; and (4) increase the minimum allowable standard deviation from 0.2 to 1.0. We tested all four changes on the training data and found that modifications #1 and #2 had minimal effects on the mean absolute residual, thus we only adopted modifications #3 and #4. The final model used a 7-day baseline, adjusted for total ED visits, and had a minimum standard deviation of 1. SAS 9.2 was used to generate the test statistic.

#### Autoregressive integrated moving average (ARIMA)

For the second method, we fit a seasonal ARIMA model, which is a modified version of the ARIMA model specified in Reis and Mandl [19], to the training data. Our final optimal model was (1,1,2)x(0,1,1)_7_ with sinusoidal cross-correlation parameters to account for seasonal trends. The model can also be expressed as:

$$\left(1-\phi_{1}B\right)\left(1-B\right)\left(1-B^{7}\right)y_{t}=\left(1+\theta_{1}B+\theta_{2}B^{2}\right)\left(1+\Theta_{1}B^{7}\right)e_{t}$$

Where *y*_*t*_ = *y*_*t*−1_, *B*^7^*Y*_*t*_ = *y*_*t*−7_, *ϕ*_1_ is the non-seasonal autoregressive coefficient, *θ*_1_ is the non-seasonal moving average coefficient Θ_1_ is the seasonal moving average coefficient, and *e*_*t*_ is the variance of the error. The non-seasonal components of our model are denoted by the first set of parameters, and include an autoregressive term with a one-day lag, a one-day differencing term, and two moving average terms with a one-day and two-day lag. The seasonal components account for weekly trends with a one-week differencing term and a moving average term with a one-week lag. In order to capture seasonal trends, we chose a longer baseline that contained multiple seasons. Thus, a three-year sliding baseline was used, forecasting one day into the future. PROC ARIMA in SAS 9.2 was used for model development and analysis.

#### Generalized linear model

We next assessed a Poisson generalized linear model (GLM). Two sliding baseline lengths were analyzed, 180 days and 56 days, the latter being similar to the adaptive regression model described by Burkom [23]. We first adjusted for long-term trends by including linear (centered to the middle of the baseline period), quadratic, and cubic terms in each model. Because of strong day of week effects in the data, we added a day of week term, and finally, a holiday indicator. In the 56-day baseline model, the cubic term was not significant, so only the linear and quadratic terms were included. Both models accounted for over-dispersion in the data, using the dscale option in SAS’s Proc GENMOD. We observed the 56-day baseline model had smaller residuals and thus was selected for our final model:

$$E\left(X_{t}\right)=\;\beta_{0}+\;\beta_{1}\left(DOW_{t}\right)+\;\beta_{2}\left(H\right)+\;\beta_{3}\left(LT\right)+\;\beta_{4}\left(QT\right)$$

Where *E(X*_*t*_*)* is the expected number of cases on day *t*, *DOW*_*t*_ is categorical day of week, *H* is a holiday indicator, *LT* is linear time trend, and *QT* is quadratic time trend. The method estimates a standardized residual which is then used as the test statistic.

#### Cumulative sum control chart

Our evaluation of CUSUM required two steps [14]. First, in order to account for systematic trends in the data, we applied the same GLM model described in the previous section. Residuals from the model were then used in a one sided CUSUM,

S(t)=max[0, S(t−1)+Y(t)−μ−k]

Where *S(t)* is the test statistic, *μ* is the target residual, and *k* is the reference interval.

We tested four baselines: 4 week, 6 week, 8 week, and 12 week. For all baseline durations, the models tended to have a higher residual on days with low syndrome counts compared to days with higher syndrome counts, i.e., overestimate low counts and underestimate high counts. Because signal strength and frequency is dependent on both baseline and reference interval, we tested four *k* values: 0.5, 0.65, 0.75, and 1. We found that as baseline length increased, signal frequency increased. The longest baseline (12 weeks) signaled often, suggesting this model might be oversensitive, whereas the shortest baseline, even with a low *k*, rarely signaled. Comparing the correlation between the standardized residuals and observed counts, we determined that the best fit model used an 8-week baseline, with a target residual μ of zero, and k set to 0.5. We used PROC CUSUM in SAS 9.2.

#### Holt-Winters exponential smoother

The Holt-Winters exponentially weighted smoothing algorithm weights a prediction at any time point (t) based on previous time points (t-n) [23]. In order to implement Holt-Winters, smoothing coefficients for level (i.e. weight of recent events vs. past events), linear trend, and seasonality must be specified. We tested weights between 0 and 1 for all inputs across a longer baseline (full training data set) vs shorter baseline (2 years). Mean squared error and R-squared were estimated for all model types. For our best fit model of the lowest mean squared error and highest R-squared, we specified weights of 0.55 for level, 0.0001 for linear trend, and 0.05 for seasonal trend, using a sliding 2-year baseline. We fit the additive Holt-Winters model using PROC FORECAST in SAS 9.2.

#### SaTScan temporal scan statistic

For our final temporal method, we evaluated the SaTScan temporal scan statistic using a discrete Bernoulli probability model, which is used by our current system, and has been previously described [2]. The current method compares the previous day, two days, or three days to a 14-day baseline, and p-values are estimated using Monte Carlo significance testing. Previous efforts at the NYC DOHMH have optimized this model to our data. Thus, we decided to keep the input parameters the same in order to evaluate other algorithms against our current methodology. We used SaTScan version 9.3.1 [27].

### Spatial and spatio-temporal methods

#### Bayesian space-time regression

We adapted the two-level Bayesian model of Corberon Vallet [22]. At the first level, within-area variability of the counts for a particular syndrome in zip code *i* and day *t* is modeled using the Poisson distribution

$$y_{it}\sim P_{0}\left(e_{it}\theta_{it}\right)$$

where the naïve expected count of visits *e*_*it*_ represents the “background” effect (calculated based on the spatial distribution of total ED visits in NYC) and *θ*_*it*_ is the unknown area-specific relative risk, which is estimated in the second level of the model.

The logarithm of the relative risk *θ*_*it*_ is decomposed in additive components representing spatial and covariate effects at the second level of the model,

$$log\left(\theta_{it}\right)=\rho +\;u_{i}+v_{i}+\;\overset{7}{\underset{d=1}{\sum }}\alpha_{d}I_{d}\left(t\right)\;+\;\overset{2}{\underset{h=1}{\sum }}\alpha_{h}I_{h}\left(t\right)$$

where *ρ* is the overall level of the relative risk, *u*_*i*_ and *v*_*i*_ represent respectively spatially correlated and uncorrelated extra variation, ${\left\{\alpha_{d}\right\}}_{d=1}^{7}$ and ${\left\{\alpha_{h}\right\}}_{h=1}^{2}$ are the day of week and holiday effects, respectively, and *I*_*d*_(*t*) and *I*_*h*_(*t*) are indicator functions which takes the value 1 if time *t* corresponds to day *d* or holiday *h*, and 0 otherwise.

The prior distributions for the intercept and uncorrelated spatial effect are uninformative and assumed to be zero mean Gaussian with variance $\sigma_{\rho }^{2}$ and $\sigma_{v}^{2}$, respectively. To borrow strength over space, the conditional autoregressive (CAR) model proposed by Besag, *et al*. [28] is used as a prior for the spatially correlated effect where the neighborhood is assumed to consist of first-order spatial neighbors defined by a common boundary. To ensure more stable estimates of day of week and holiday parameters, the prior distributions are informative and assumed to be normally distributed with mean and variance of a sample (four weeks apart to reduce correlation) of estimates of these parameters when the model was run with a 6-month baseline over a 3 year period.

A 15-day moving baseline period was chosen, in part because of computational restrictions, as increasing length of baseline substantially increases runtime and local memory requirements. Another factor that influenced this decision was the recognition that longer baselines smooth over possible shifts in relative risk and diminish the ability of the method to detect abrupt increases in syndrome visits. Inputs were combined in a hierarchical model to estimate ZIP code-specific expected visits, and alerts were generated on days for which observed values within a ZIP code were significantly higher than expected values as measured by a Bayesian diagnostic. We used a combination of SAS 9.2, R 3.0, and WinBUGS14.

#### Generalized linear mixed model

Our generalized linear mixed model (GLMM) was similar to one proposed by Kleinman, et al [21] and was developed in consultation with the project’s Advisory Board. The R software’s “glmmPQL” model (GLMM model using Penalized Quasi-Likelihood) was applied, specifying family = “Poisson”. The model included fixed effect terms for total ED visits, season, and day-of-week and a random intercept term for ZIP code. We tested two baselines: the first based on the 56-day sliding baseline GLM model described above and the second on a longer 6-month baseline. As the ZIP code-level daily counts were low, and in some ZIP codes extremely low, the 6-month baseline was considered to be more appropriate. The model could be expressed as:

$$log\left[\mu \right]=\beta_{0}+\beta_{1}\left(ED\right)+\beta_{2}\left(DOW,df=6\right)+\beta_{3}\left(D,df=8\right)+\overset{183}{\underset{z=1}{\sum }}b_{z}$$

Where *ED* is the total number of ED visits, *DOW* is day of week, *D* is date, and *b* is the random intercept in ZIP code *z*. A signal or an aberration from the expected was based on residuals outputted from the GLMM model. For each ZIP code, the mean and standard deviation of the residuals for the same day of week during the 6-month baseline period were used to compute threshold cutoffs. Alternate cutoff options were explored and signals were generated when the current day’s ZIP code residual was found to be larger than *n* = 1, 2, 2.5, 3, 3.5, 4, 4.5, 5, 5.5, 6, and 10 standard deviations from the mean.

#### SaTScan space-time permutation scan statistic

A prospective space-time scan statistic was evaluated using the space-time permutation probability model in SaTScan version 9.3 [11]. The scanning window in the space-time permutation can be thought of as a cylinder, with the base representing the geographic area of analysis, and the height representing time. The cylinders are then iterated over the ZIP code centroids, with the base radius gradually expanding to a user-determined maximum (in our case 50% of syndrome visits in the city) and base height ranging from 1 day to a maximum (we limited to 30 days). These inputs were selected based on the size and length of our simulated outbreaks. To account for multiple testing, SaTScan shuffles the temporal and spatial characteristics and creates many random permutations of the reorganized data (we limited to 999 permutations). We used standard unadjusted p-values to account for repeated analyses, as recommended by Kulldorff and Kleinman [29]. Cluster significance is then estimated using Monte Carlo hypothesis testing from the simulated datasets.

#### SaTScan spatial scan statistic

Finally, we evaluated the SaTScan spatial scan statistic using the discrete Poisson probability model in SaTScan 9.3, our current methodology, which we’ve described previously [2]. The spatial scan statistic compares visits from the previous day to a user-defined baseline (we used 14 days) in each geographic area of analysis. Similar to the space-time permutation described above, the geographic window can be thought of as a circle, which is then iterated over ZIP code centroids in expanding circle size (we set to a maximum of 20% of syndrome cases in the city). The temporal window is limited to one day. Baseline length and spatial window were based on our current input parameters. Similar to the other SaTScan analyses, we determined the statistical significance of the clusters using Monte Carlo testing set at 999 replications.

### Candidate method testing

Models were first tested on the training dataset to determine model baseline, final model variables, and fine tune model parameters. Once the models were optimized, we then tested each of the models on the datasets with the simulated injects. The following section describes the metrics for comparing model performance.

#### Comparing method performance

Each method was run on the spiked datasets from the beginning to the end of the two year period. Thresholds were chosen for each method to give a range of specificities in order for all methods to be compared on a receiver operator characteristic (ROC) curve. The methods were evaluated on two categories of criteria: (1) proportion of spiked injects correctly and incorrectly detected and (2) the difficulty in development, implementation, and interpretation of the method. For the first part of the evaluation, we estimated sensitivity, specificity, positive predictive value, timeliness, and the proportion of inject days in which the method signaled (what we will call coherence). These measures are commonly used as performance metrics in syndromic surveillance literature [12,13,15,17,18]. For the purposes of calculating these measures, we defined a detected inject as a signal on any day in a spiked dataset for temporal methods, and for the spatial methods, a signal in any of the spiked ZIP codes within an inject. In other words, an inject was successfully detected if a temporal method caught at least one spiked day or a spatial method caught at least one spiked ZIP code in one spiked day. Conversely, non-injects were defined as a day without a signal for the temporal methods. For the spatial methods, non-injects were defined as no signals in any of the 183 daily ZIP codes. The performance measures are defined as sensitivity (the number of injects detected, i.e., true positives) divided by all injects), specificity (the measure of correctly identifying the non-injects; the number of non-injects, i.e., true negatives, divided by all non-injects),positive predictive value (the number of correctly identified injects divided by all signals; his is a measure of the probability of a signal being a true signal),timeliness (signal timeliness was measured as [1-(first day of signal/number of days in outbreak)];values range from 0 to 1, with 1 indicating the best timeliness), coherence (the percentage of inject days in which the method signaled, regardless of size of cluster detected).

All evaluation analyses were conducted using SAS version 9.2, R (version 2.13.0, R Development Core Team), and WinBUGS (version 1.4.3, Cambridge Institute of Public Health, Cambridge, UK). For the second part of the evaluation, we noted each method’s implementation into the chosen statistical software, programming time, run time, and the ease of use of the method, taking into account the familiarity of the programming package, the statistics used, and interpretation of the output. At the time of the study, SAS was the most widely used software at the NYC DOHMH and thus, most widely known to analysts. However, we considered R and other statistical software if there were specific methods or analyses that were difficult to implement in SAS.

## Results

### Performance evaluation of temporal methods

**Fig 2:**
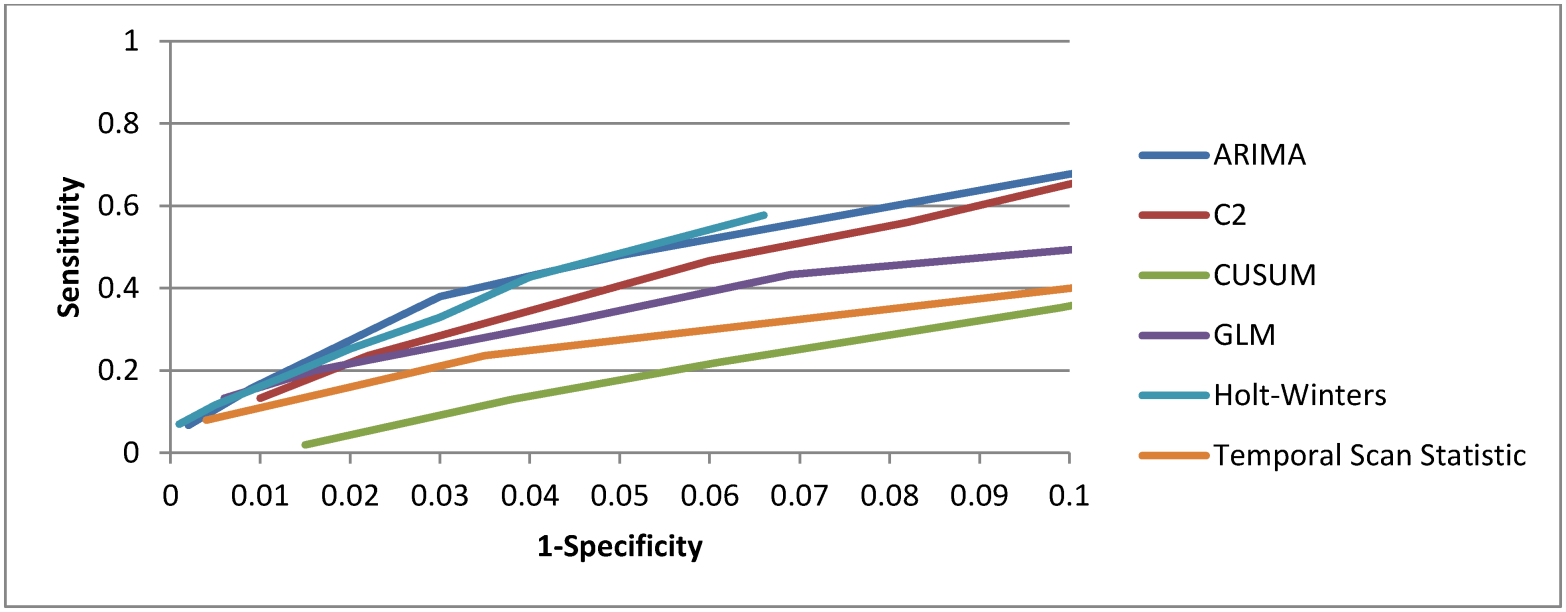
Receiver operator characteristic (ROC) curve for tested temporal methods.

We show the overall performance of the temporal methods in Fig 2. We ran each temporal method on the 300 spiked citywide datasets. Across all inject types and sizes, the Holt-Winters detected the most injects (19%), followed by the ARIMA (16%), GLM (15%), modified C2 (13%), temporal scan statistic (11%), and CUSUM (<2%), at a specificity of 0.95. A detailed breakdown of method performance by epidemic curve and magnitude, at a specificity of 0.95, is given in Table 5. This specificity was chosen because this is the minimum at which we would trigger further investigation into a signal.

**Table 5:** Metrics for temporal methods for citywide injects at specificity 0.95.

		Small sized injects (1 SD)			Medium sized injects (2 SD)			Large sized injects (3 SD)		
		Sens	PPV	Time	Sens	PPV	Time	Sens	PPV	Time
**ARIMA**	**Single day spike**	0.20	0.01	N/A	0.55	0.02	N/A	0.95	0.03	N/A
**C2**		0.20	0.01	N/A	0.55	0.02	N/A	0.75	0.02	N/A
**CUSUM**		0.00	0.00	N/A	0.00	0.00	N/A	0.10	0.01	N/A
**GLM**		0.00	0.00	N/A	0.45	0.01	N/A	0.75	0.02	N/A
**HW**		0.30	0.01	N/A	0.65	0.02	N/A	0.95	0.03	N/A
**TSS**		0.05	0.01	N/A	0.20	0.01	N/A	0.35	0.01	N/A
**ARIMA**	**Point source**	0.38	0.01	0.70	0.33	0.01	0.84	0.65	0.02	0.72
**C2**		0.28	0.01	0.68	0.32	0.01	0.68	0.52	0.03	0.63
**CUSUM**		0.07	0.01	1.00	0.03	0.01	0.83	0.13	0.05	0.63
**GLM**		0.23	0.01	0.83	0.28	0.02	0.83	0.45	0.03	0.67
**HW**		0.27	0.01	0.69	0.33	0.02	0.78	0.67	0.02	0.73
**TSS**		0.22	0.02	0.78	0.27	0.01	0.54	0.28	0.02	0.57
**ARIMA**	**Propagated**	0.45	0.02	0.61	0.40	0.02	0.51	0.55	0.03	0.51
**C2**		0.40	0.02	0.66	0.30	0.01	0.45	0.50	0.02	0.46
**CUSUM**		0.20	0.04	0.88	0.05	0.03	0.29	0.15	0.06	0.73
**GLM**		0.15	0.02	0.59	0.25	0.01	0.70	0.35	0.03	0.72
**HW**		0.45	0.02	0.69	0.55	0.02	0.67	0.65	0.03	0.59
**TSS**		0.25	0.02	0.67	0.45	0.03	0.70	0.55	0.04	0.51

The Holt-Winters also had the highest sensitivity across the majority of inject types compared to the other methods. Across all methods, sensitivity was highest among the largest outbreaks. There was no method that was consistently the fastest in time to detection. All the methods showed poor PPV, never exceeding 6%. Coherence was low for all methods, ranging from less than 1% to-8%. We also ran the modified C2 on the injected data using a 14-day and 28-day baseline, as recommended by Tokars [12], and found no differences in performance compared to the 7-day baseline (data not shown).

### Performance evaluation of spatial and spatio-temporal methods

**Fig 3:**
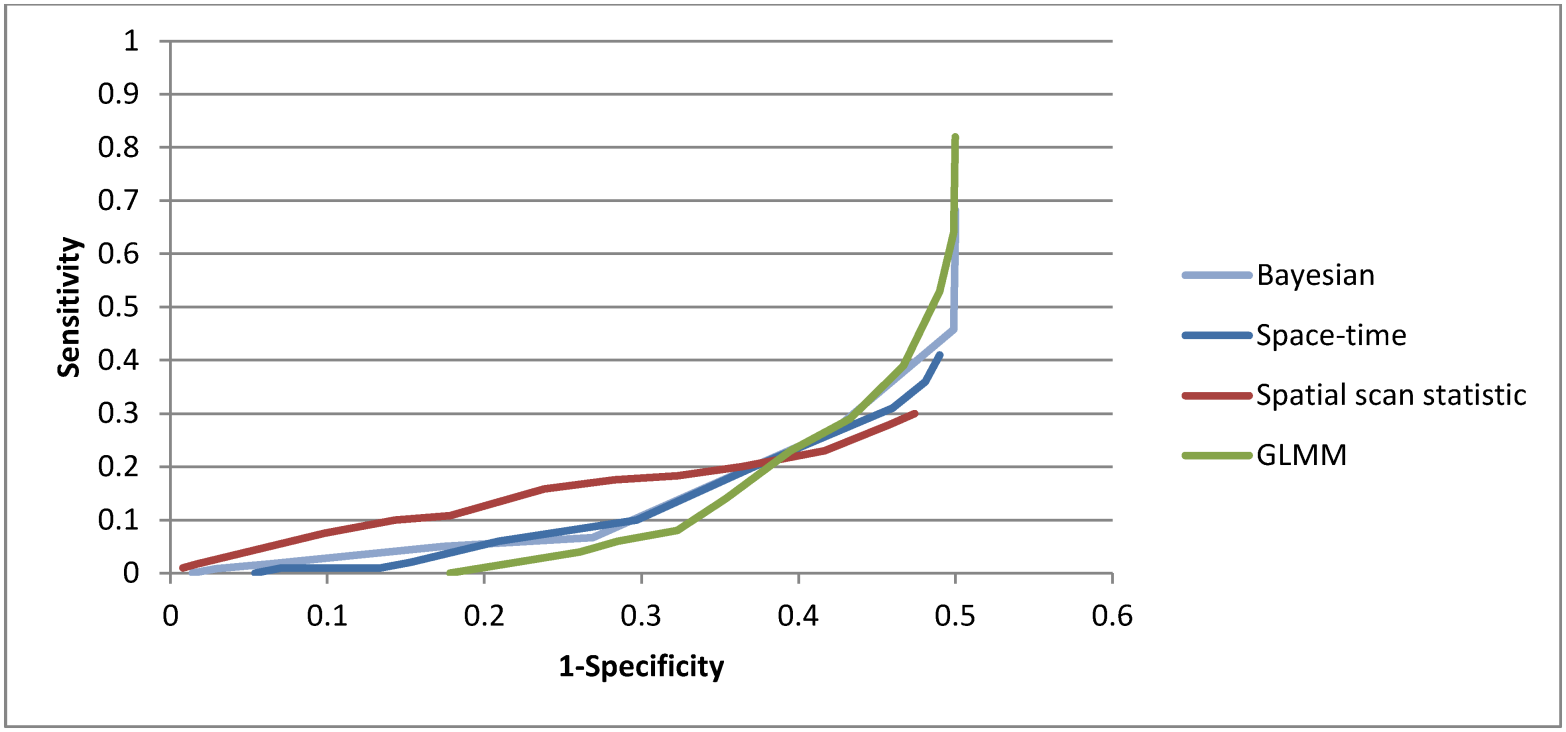
ROC curve of spatial and spatio-temporal methods, all outbreak types.

In Fig 3, we show the ROC curve for the spatial/spatio-temporal methods across all outbreak types. At a specificity of 95%, the spatial scan statistic detected 3% of all outbreaks, the Bayesian regression detected 2%, and the GLMM and space-time permutation scan statistic detected none.

**Fig 4:**
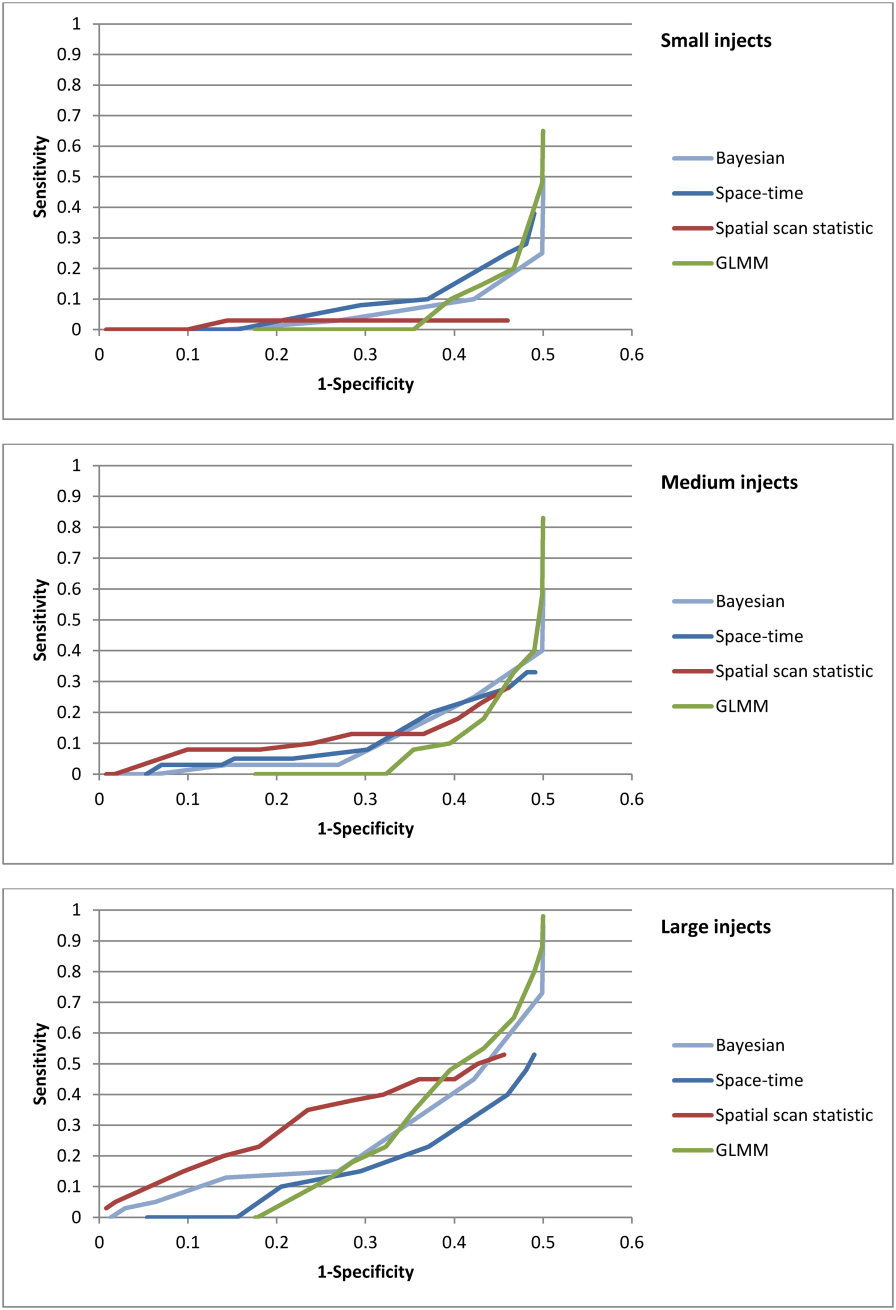
ROC curves of spatial and spatio-temporal methods, by inject magnitude.

Method performance improved with larger (3 SD above baseline) injects (Fig 4). At a specificity of 95%, none of the methods detected an outbreak of less than 3 SD above baseline and at 3 SD, only 8% of injects were detected by the spatial scan statistic and 4% by the Bayesian regression. The GLMM and space-time permutation scan statistic did not detect any outbreaks at the 95% specificity level.

PPV was essentially zero for all the methods, regardless of inject size. With regard to timeliness of inject detection, the spatial scan statistic had the shortest time to detection (0.63) followed by the Bayesian regression (0.54). Coherence never exceeded more than 1% for any of the methods.

**Fig 5:**
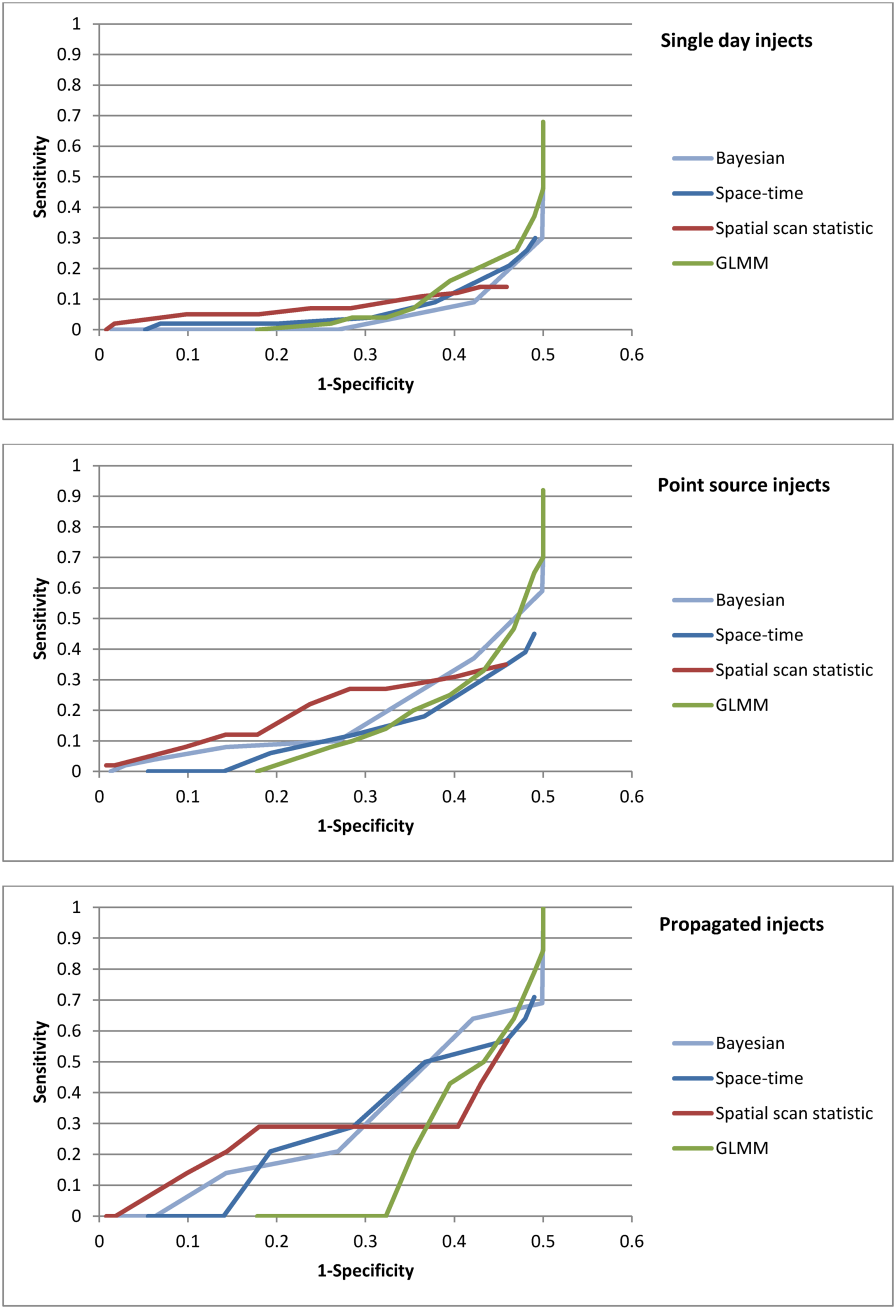
ROC curves of spatial and spatio-temporal methods, by inject type.

We then examined performance by inject type (Fig 5). The spatial scan statistic found 2% of single day injects, at a specificity of 95%, while the other methods detected none. Among point source outbreaks, the Bayesian regression found 4% and the spatial scan statistic 2%. Only the spatial scan statistic detected propagated injects (7%). PPV for all the methods was low (less than 1% of ZIP code level signals were true signals).

### Practice-based evaluation of the methods

Ultimately, disease detection methods can only be used if they can be successfully implemented, maintained, and updated in practice. For syndromic surveillance, this is usually at the local public health setting. As such, a thorough evaluation would acknowledge the importance of practice-based considerations.

Table 6 shows the results of the practice-based metrics. The temporal methods were programmed in SAS 9.2 and SaTScan. The programming time varied based on the method and ranged from low of 3 days for the GLM and temporal scan statistic to a high of 3 weeks for ARIMA. The programming time, however, did not reflect the amount of time required for the analyst to understand and develop the base model, which depending on the model, ranged from weeks (e.g., GLMM) to months (e.g., Bayesian). The run time (an average of three runs) for the temporal methods never exceeded more than 4 seconds of processing time. The spatial/spatio-temporal methods were coded in R, WinBUGS, and SaTScan. Programming time ranged from 3 days (spatial scan statistic and the space-time permutation) to 10 weeks (Bayesian). Run time ranged from 6 seconds (spatial scan statistic) to 2 minutes (Bayesian).

**Table 6:** Programming metrics for tested methods.

Method	Software	Programming time	Run time (sec)	Ease of use
**Temporal methods**				
ARIMA	SAS 9.2	3 weeks	00:01	Significant amount of testing needed for the final model inputs; also, it is a method that has not frequently been used at DOHMH so there was a learning curve; its description in the literature [19] and actual coding of the method were straightforward
C2	SAS 9.2	1 week	00:01	One of the most commonly used methods; easy to understand; easy to code and well documented in the literature [26]
CUSUM	SAS 9.2	1 week	00:01	Commonly used method; determining inputs to the CUSUM model was the most difficult part; otherwise, easy to code and well documented [14]
GLM	SAS 9.2	3 days	00:01	Commonly used model; accessible to most analysts to understand, code, and troubleshoot [23]
Holt-Winters	SAS 9.2	2 weeks	00:01	Experience was similar to the ARIMA where significant time went into developing the model specifications; the method has not been frequently used but is accessible and not difficult to understand [23]
Temporal scan statistic	SaTScan	3 days	00:04	Much of the programming is done already in the SaTScan program [27], so analysts only needed to define parameters; if called from SAS or R, will need to write programs to define the parameters and run macros
**Spatial/spatio-temporal methods**				
GLMM	R	1 week	0:50	Moderately difficult to program the model and output, knowledge of regression models needed [21]
Bayesian	R, WinBUGS	10 weeks	2:00	Highly difficult; extensive knowledge of several statistical packages and advanced statistics is required to understand and implement [22,30]
Spatial scan statistic	SaTScan, R	3 days	0:06	Much of the programming is done already in the SaTScan software [27], so analyst only needed to define parameters; some knowledge of spatial epidemiology is needed
Space-time permutation	SaTScan, R	3 days	0:12	Same as spatial scan statistic [27]

## Discussion

### Summary and main findings

This study was undertaken to compare and evaluate the performance of our current aberration detection methods to alternative methods published in the literature. Our goal was to determine the best temporal and spatial/spatio-temporal methods applicable to our data, using a combination of performance and practice-based metrics. Furthermore, it was important to us to build and implement the methods to reflect how we would use them in daily practice. While previous method evaluations have focused on performance, with good reason, we believe it important to determine the work load burden and level of expertise required in implementing these various methodologies in a syndromic system.

Overall we found the spatial/spatio-temporal methods, including our current method, did not work well in detecting small simulated injects at the ZIP code level. Sensitivity improved with increased magnitude of injects, which aligns with other evaluations of aberration detection methods for syndromic surveillance [14,15,19,31]. PPV for all methods was negligible, suggesting that when these methods signaled, it was rarely the result of the simulated injects, but more likely from a number of things such as data anomalies or errors, an actual outbreak, or quite possibly just a false signal. We also found that performance differed between single day spikes, point source injects, and propagated injects for both spatial/spatio-temporal and temporal methods. Based on our findings, the spatial scan statistic worked best for detecting spatial clusters, though its performance was not much better than the other tested methods. The GLMM model used in this evaluation needs refinement to reduce the large number of false positives. Further development of this model to include information about spatial proximity between ZIP codes—to aid in the identification of spatial clusters—would also be beneficial.

### Other findings and comparison to other studies

Performance of our tested methods differed by the shape and duration of the injections, which was consistent with some [14,18] but not all [15] studies. However, we should note that published evaluations of syndromic surveillance systems tend to focus primarily on temporal methods for outbreak detection [14,15,23,32]. Sensitivity was better for point source and propagated injects, and in general, increased as inject duration increased. This was expected given there was more opportunity for detection with longer injects. In general, the ability of these methods to detect a spike in cases in the early stages of an inject was good, with the exception of the spatial scan statistic detecting propagated injects. We did not estimate timeliness and coherence for single day spikes because there was only one day to measure. For the temporal methods we tested, we found one-day single-day spikes to be the most commonly detected injects when inject size was large and propagated injects when inject size was small. An evaluation of temporal methods by Jackson, et al [15] suggests the GLM should have outperformed the C2 across all inject sizes and distributions, though we found the opposite. This difference is likely due to the modifications we made to the C2, as suggested by Tokars, et al. [12]. Similarly, Fricker, et al., [14] showed a CUSUM with a 56-day sliding baseline outperformed a C2 in several scenarios of different inject magnitudes and durations. Our findings suggest a modified C2 performed better than the 56-day sliding baseline CUSUM. One recent study expressed concern with use of the prospective scan statistic in SaTScan, citing issues with adjusting for previous analyses [33], though further discussion has emphasized the appropriate use of recurrence intervals based on standard unadjusted *p*-values [29], which was how we dealt with repeated analyses. PPV was low across all methods and inject types, likely due to our use of actual syndromic data, which contained unknown outbreaks.

### Generalizability to other health departments and lessons learned

We chose to use injected simulated spikes in real datasets for this evaluation because we believed that the performance would most accurately reflect real experience, specifically with the temporal and spatial patterns found in our data. However, our evaluation approach likely penalized these methods by ignoring real outbreaks in the data. There is much debate about whether simulated data is a better alternative, especially if they are made to reflect real data as much as possible [34]. Future work may want to include testing of both actual and synthetic syndromic baseline data, as well as actual and simulated outbreak events, in parallel, as recommended by Fricker [34]and Unkel and colleagues [35].

We ran all spatial/spatio-temporal methods on ZIP code level data, which was the finest spatial granularity available for analysis in the DOHMH syndromic surveillance system. While analyzing small spatial units is ideal from a public health perspective given potential disease outbreaks can be pinpointed with reasonable precision, from the analytic viewpoint, ZIP code-level counts can be sparse and vary widely. Thus, we would recommend exploring a range of spatial granularities and perhaps choosing a spatial scale for which syndrome counts or rates are less variable.

Typically when the NYC syndromic system identifies a cluster at the hospital or ZIP code-level, the syndromic analyst follows up by “eye-balling” the visit-level information for patients in the cluster to check for any anomalous patterns in age, time of visit, or disposition. Clusters that look suspicious or unusual are followed up by contacting ED staff where the cluster occurred. Due to high false positives, it is essential that protocols provide guidance on which signals need follow-up or careful observation on subsequent days. The application of sophisticated models cannot alone determine a cluster or an outbreak. Proper cluster identification requires an experienced analyst with a good understanding of syndromic data and data quality issues, as well as an understanding of statistics and the models being applied.

An evaluation of methods would not be complete without a comment on the quality of data used in the models. Syndrome definitions are based on querying the chief complaint, an unstructured free-text field, for key terms. These key terms can include clinical or lay person words and phrases. Being that chief complaint data is not standardized and highly dependent on hospital coding practices, using sophisticated modeling techniques for aberration detection at smaller spatial resolutions is very challenging. Therefore, we highly recommend that before building any statistical model, health departments explore the completeness and quality of their chief complaint syndromic data.

Finally, regarding the ease of adapting these models to our syndromic data, we found that some models (e.g., Bayesian) were more complicated, required additional training, and therefore took longer to develop and implement. This is an important consideration when deciding whether to test alternative models.

### Limitations

This study has several limitations. Only published methods were selected, and of those, only a few were able to be evaluated. Thus there potentially could be better performing methods we did not evaluate. Also, we used actual syndromic data for our analytic baseline. Detection of unknown outbreaks or other data anomalies in the baseline data is counted as a false positive, thereby leading to lower specificity and positive predictive values. However, a common argument against using purely synthetic data is that it does not capture all of the nuances and variation of ED visit data. Furthermore, because of the computational intensity of running these methods spatially, we were able to run only a relatively small number of simulations, thus limiting our understanding of how methods performed over a wide range of inject sizes. For this study, our focus was on detection of small injects of disease. As a consequence, the majority of injects were small and thus many of the methods had difficulty in determining the signal from the noise. The spatio-temporal methods used in this study take very different approaches to identifying signals and therefore a direct comparison of model performance is difficult. The spatial scan statistic and space-time permutation were the only spatial methods that identified “clusters,” i.e., reported signals for a group of neighboring ZIP codes. The other models treated ZIP codes as being independent of each other and did not include any information about how ZIP codes might be spatially related. This information could be included in the models described, however this wasn’t attempted due to its complexity.

### Recommendations and conclusions

In summary, our evaluation suggests the models we tested did not perform well in detecting temporal and spatial clusters of cases. We chose to analyze injects at the ZIP code level primarily because this is the smallest spatial granularity reported to our system. But we suggest other jurisdictions analyze data at a spatial granularity that makes sense to them (which may or may not be at the ZIP code level). As data quality improves as hospitals move towards standardized electronic health record and data transmission practices, these methods might be more successful in detecting aberrations. We are currently using the space-time permutation scan statistic in parallel with the spatial scan statistic in our system and will prospectively evaluate the frequency and makeup of signals from the two methods. We found the Bayesian regression to show promise in detecting injects and plan to compare this method with the scan statistics, while addressing some of the limitations of the current study.

While syndromic surveillance was set up for early detection of large-scale bio-terrorism related events, identifying disease clusters continues to be a challenge. Nonetheless, syndromic systems have shown the ability to track seasonal disease trends, such as influenza and norovirus, and provide valuable real-time situation awareness during evolving events (e.g., hurricanes, heat emergencies). It can be certain that syndromic surveillance will continue to provide timely and useful information, once an event has occurred, beyond just finding aberrations in count data.
